# How to Avoid Colds

**Published:** 1885-08

**Authors:** 


### How to Avoid Colds.

Physiologists have said that if a few drops of the blandest fluid in nature are injected into a blood-vessel against the current, death is an instantaneous result.

Millions of canals or tubes from the inner portions of the body, open their little mouths at the surface, and through these channels, as ceaseless as the flow of time, a fluid containing the wastes and impurities of the system is passing outwards, and is emptied out on the skin; ordinarily, it is so attenuated, so near like the air, that it can not be seen with the naked eye, but extraordinarily, under the influence of increased natural or artificial heat, as from exercise or fire, this fluid is more profuse, and is seen and known as "the sweat of the brow"—perspiration.

This fluid must have exit or we die in a few hours. If it does not have vent at the surface of the body, it must have some internal outlet. Nature abhors shocks as she does a vacuum. Heat distends the mouths of these ducts, and promotes a larger and more rapid flow of the contained fluid: on the other hand, cold contracts them, and the fluid is at first arrested, dams up and rebounds. If the purest warm milk, injected against the current of the blood, kills in a moment, not from any chemical quality, but from the force against the natural current, there need be no surprise at the ill-effects of suddenly closing the mouths of millions of tubes at the same instant, causing a violence at every pin-head surface of the body. If these mouths are gradually closed, nature has time to adapt herself to the circumstances by opening her channels into the great internal "water-ways" of the body, and no harm follows. Hence the safety of cooling off slowly after exercise or being in a heated apartment, and the danger of cooling off rapidly, under the same circumstances, familiarly known by the expression "checking the perspiration."

The result of closing the pores of the skin is various according to the direction the shock takes, and this is always to the weakest part; in the little child it is to the throat, and there is croup or diphtheria; to the adult it is to the head, giving catarrh in the head or running of the nose; to the lungs, giving a bad cold, or, if very violent, causing pneumonia or inflammation of the lungs themselves; or pleurisy, inflammation of the covering of the lungs; to the bowels causing profuse and sudden diarrhœa, or to the covering of the bowels, inducing that rapid and often fatal malady known as peritoneal inflammation; if the current is determined to the liver, there is obstinate constipation, or bilious fever, or sick headache. Hence, a "cold" is known by a cough, when perspiration is driven inward, and is directed to the lungs; by pleurisy, when to the lining of the lungs; by a sick headache or bilious fever, when to the liver, etc.; diarrhœa or constipation when to the bowels and liver.

To avoid bad colds, then, it is only necessary to avoid closing the pores of the skin, either rapidly, by checking perspiration, or slowly, by remaining still until the body is thoroughly chilled, that is, until the pores are nearly or entirely closed by inaction in a cold atmosphere or room. In the matter of health, these suggestions are of incalculable importance.

